# IRG1/itaconate increases IL-10 release to alleviate mechanical and thermal hypersensitivity in mice after nerve injury

**DOI:** 10.3389/fimmu.2022.1012442

**Published:** 2022-10-13

**Authors:** Qingyu Sun, Tingting Hu, Yurui Zhang, Xiaotong Wang, Jing Liu, Wen Chen, Chao Wei, Dianxin Liu, Weihua Wu, Ting Lan, Yumeng Ding, Zhaoli Luo, Meng Liu, Danmin Shen, Zhongnan Xiao, Liye Hu, Miaoyi Pang, Yiran Ma, Lei Shi, Peipei Wang, Jiannan Zhang, Qian Li, Fei Yang

**Affiliations:** ^1^ Department of Neurobiology, School of Basic Medical Sciences, Capital Medical University, Beijing, China; ^2^ Department of Anesthesiology, Chang Hai Hospital, Naval Military Medical University, Shanghai, China; ^3^ Department of Biochemistry and Molecular Biology, School of Basic Medical Sciences, Capital Medical University, Beijing, China; ^4^ School of Basic Medical Sciences, Capital Medical University, Beijing, China; ^5^ Advanced Innovation Center for Human Brain Protection, Capital Medical University, Beijing, China; ^6^ Key Laboratory of Cancer Invasion and Metastasis Research, Capital Medical University, Beijing, China

**Keywords:** itaconate, interleukin-10, neuropathic pain, tricarboxylic acid cycle, IRG1

## Abstract

Inflammation plays an important role in the occurrence and development of neuropathic pain. Immune-responsive gene 1 (IRG1) decarboxylates *cis*-aconitate to produce itaconate in the mitochondria. Itaconate serves as an immunomodulator of macrophages and represses inflammation in infectious diseases. Recently, a study showed that an itaconate derivative inhibits neuroinflammation and reduces chronic pain in mice. However, the function and molecular mechanisms of endogenous itaconate in neuropathic pain have not been fullyelucidated. In this study, the content of itaconate in the ipsilateral spinal cord after nerve-injured mice was detected with mass spectrometry. The *Irg1^-/-^* mouse was constructed to determine the role of endogenous itaconate in the chronic constriction nerve injury (CCI) model. The analgesic effect of exogenous itaconate was assessed with intraperitoneal and intrathecal administration in both male and female CCI mice. The spinal application of 4-OI also reduced the evoked responses of wide dynamic range neurons in CCI mice. The potential analgesic mechanism of itaconate was explored through molecular biology experiments and verified in Interleukin *(IL)-10^-/-^* mice. We found the levels of itaconate and IRG1 in the spinal cord significantly increased after CCI. *Irg1* deficiency aggravated the mechanical and heat hypersensitivity, while the exogenous administration of the itaconate derivative 4-OI alleviated the neuropathic pain in male and female CCI mice. Mechanistically, the treatment of 4-OI increased the level of IL-10 and activates STAT3/β-endorphin pathway in the spinal cord, and the analgesia effect of itaconate was impaired in *IL-10^-/-^* mice. Finally, we showed that the upregulation of IL-10 induced by 4-OI was mainly from spinal neurons through Nrf2 pathway. This study demonstrated the analgesic effect of endogenous and exogenous itaconate in the neuropathic pain model, suggesting that the spinal IL-10/STAT3/β-endorphin pathway might mediate the analgesia effect of itaconate.

## Introduction

Neuropathic pain is caused by damage or diseases of the somatosensory system (1, 2). Evidence indicates that the inflammatory response plays a vital role in the development of hyperalgesia in neuropathic pain (3–5). At peripheral, infiltrated immune cells release mediators to sensitize and activate the nerve endings of nociceptor neurons, and the generated action potentials are transduced to the spinal cord through the dorsal root ganglia (6, 7). In the dorsal horn of the spinal cord, nociceptive afferent neurons (presynaptic) release glutamate, ATP, and chemokines from their peripheral synapses, which mediate signal transmission to second-order postsynaptic neurons and transmit signals to the brain (6, 8). Meanwhile, microglia, astrocytes, and even T cells produce pro-inflammatory cytokines and growth factors, which act on pre-synaptic and post-synaptic nerve terminals to increase signal transmission and mediate central pain sensitization (6, 9). Therefore, the inflammatory process is of great significance to the occurrence and development of neuropathic pain.

Itaconate is a metabolite of the tricarboxylic acid (TCA) cycle and is produced during the activation of macrophages (10). The *cis*-aconitate decarboxylase immune response gene 1 (IRG1) is encoded by *Acod1* (refers to as *Irg1* here) and is the enzyme responsible for itaconate production (11, 12). Itaconate was first discovered in 1836 (13). Until 2018, studies discovered that itaconate is an effective immunomodulator and has anti-inflammatory effects (13, 14): in the later stages of inflammation, IRG1 overexpression causes itaconate accumulation and itaconate inhibits the succinate dehydrogenase to limit the production of mitochondria reactive oxygen species (15–17). In addition, itaconate alkylates the cysteine residue of kelch like ECH associated protein 1 (Keap1), resulting in nuclear translocating of nuclear factor erythroid 2-related factor 2 (Nrf2) and promoting the expression of anti-inflammatory and antioxidant genes (18). Itaconate also plays the roles as the regulator of type I interferon (IFN) and inhibitor of NLR family pyrin domain containing 3 (NLRP3) inflammasome in macrophages (19, 20). More recently, a study showed that dimethyl itaconate, an itaconate derivative, inhibits neuroinflammation and reduces chronic pain in mice (21). Although the immunomodulatory properties of itaconate have been explored comprehensively in macrophages, the analgesic effects of endogenous itaconate in neuropathic pain has not been studied.

Interleukin (IL)-10 is a well-characterized cytokine with anti-inflammatory and analgesic functions (22–25). IL-10 inhibits the pain response by inhibiting inflammatory factors production (23, 24, 26–28). Although type I IFN-driven IL-10 inhibits itaconate synthesis in lipopolysaccharide-treated macrophages (29), whether itaconate regulates the expression and function of IL-10 in neuropathic pain remains unknown. In this study, we demonstrated for the first time that itaconate increased after peripheral nerve injury in the spinal cord and contributed to the endogenous analgesia in the chronic constriction nerve injury (CCI) model. Meanwhile, administration of the itaconate derivative 4-octyl itaconate (4-OI) by intraperitoneal and intrathecal injection dose-dependently alleviated mechanical and heat hypersensitivity in the CCI mice. The results also suggested that the spinal Il-10 pathway might be involved in the analgesic effect of itaconate.

## Materials and methods

### Animals

The animal study was reviewed and approved by the capital medical university animal care and use committee. All experiments were carried out with appropriate anesthesia methods to minimize the suffering of experimental animals. The experimental animals were bred in the specific-pathogen free (SPF) animal facility in the Animal Department of Capital Medical University. The mice were kept in a cage facility where they could eat and drink freely (22 ± 2°C, 55 ± 5% humidity, 12 hours of light: 12 hours of darkness).

The transgenic mice used in this project were all C57BL/6J (wildtype, WT) backgrounds and 6-8 weeks old. The WT, *Irg1* ^-/-^ and *IL-10* ^-/-^ mice were obtained from Charles River Experimental Animal Technical Co., Ltd., Southern Model Organisms (Shanghai, China) and Beijing Biocytogen Biotechnology Co., Ltd. respectively.

### Chronic constriction nerve injury (CCI) model

Mice were anesthetized under the induction of isoflurane (1.0-1.5% in 100% O2, 0.5 L/min). As reported previously (30), hemostatic forceps were used to stimulate the hind paws of the mice with appropriate strength. The operation was performed while the mice did not respond to the stimulation. The left side sciatic nerve was exposed and separated from the surrounding muscle. Three loose ligations were tied with a 1 mm interval using No. 6-0 surgical thread (Cheng-He, China). When the hind paws on the operation side of the mice twitched slightly, the tightness of the ligation was the most suitable. Sham-operated mice were exposed the sciatic nerve in the same way without ligations.

### Behavioral testing

The method of von Frey and Hargreaves was used to detect changes in the hyperalgesia threshold of mice. Behavior tests were performed by investigators blind to the experimental settings.

The von Frey test: Before detecting the basic threshold, place the mice in an acrylic box to adapt for three days (30 min/day). After the mouse is quiet, use von Frey hair (NC12775, North Coast Medical Company, USA) to press vertically, until the filament is bent, starting from 0.16 g, and use the up and down method to perform the experiment. When the mouse exhibits the behavior of lifting, retracting, and licking, it is considered to have a pain response.

Hargreaves test: Before the baseline threshold was measured, the mice were placed in a box on a glass platform for three days (30 minutes per day) to adapt to the environment. After the mice are quiet, they were tested using the Hargreaves device (37370 Ugbasili, Italy). The heat was used to stimulate the hind paws of mice, and when the mice show the behavior of lifting, retracting, and licking, it is considered to have a pain response. To avoid tissue damage, set the thermal stimulation time to 25 seconds.

We calculated maximum possible effect (MPE) values to establish a dose-response function for PWT and PWL data. The MPE value for reducing mechanical hyperalgesia and thermal hyperalgesia was calculated by the following formula: MPE (%) = [1 − (Preinjury PWT or PWL− Postdrug PWT or PWL)/(Preinjury PWT or PWL − Predrug PWT or PWL)] × 100. PWT: paw withdrawal threshold; PWL: paw withdrawal latency.

### Intrathecal injection

Experimental mice were induced anesthetized with isoflurane and fixed. The operator hold a microsyringe and inserted the needle vertically at the level of the anterior superior iliac spine of the mouse. The needle insertion point was located in the space between the L5-L6 vertebral segments of the mouse. When the mouse tail shook slightly, 10 μL 4-OI solution was slowly injected. The needle was retained for 1 min and then was withdrawn to prevent the injected liquid from flowing out.

### Liquid chromatography-mass spectrometry (LC-MS)

Detection of TCA metabolites was supported by Lipidall Technologies Company Limited, China. Itaconic acid and TCA cycle metabolites were extracted from mouse spinal cord tissue using acetonitrile: water (1:1) and derivatized using 3-nitrophenylhdyrazones. The content of itaconic acid and TCA was analyzed using Jasper HPLC coupled to a Sciex 4500 MD system. Briefly, itaconic acid and TCA were separated on a Phenomenex Kinetex C18 column (100 x 2.1 mm, 2.6 µm) using 0.1% formic acid in water as mobile phase A and 0.1% formic acid in acetonitrile as mobile phase B. d4-succinic acid, d4-citric acid, d3-malic acid, 13C-3-lactic acid, d3-pyruvic acid, d4-fumaric acid used as quantitative internal standards were purchased from Cambridge Isotope Laboratories.

### Cell culture

BV2 cell culture medium: DMEM F12 (Gibco, C11330500BT) + 10% FBS (Vistech, SE100-B), N2A cell culture medium: DMEM (Gibco, C11995500BT) + 10% FBS (Vistech, SE100-B). Cells were grown in 12-well plates. 2x10^5 cells were seeded per well and cultured in a 37°C, 5% CO2 incubator. Cells were stimulated with LPS (100 ng/ml). According to the experimental results of other members of our research group, 4-OI (120µM) and ML385 (2µM) were selected to treat the BV2 cell line; 4-OI (10µM) and ML385 (5µM) were selected to treat N2A cell line. The cell culture medium was then collected for ELISA experiments.

### Enzyme-linked immunosorbent assay (ELISA)

The changes in interleukin (IL)-6, IL-1β, Tumor necrosis factor (TNF)-α, and IL-10 protein levels in the L3-L5 spinal cord segments of mice were detected with ELISA kits (Mouse IL-6 Quantikine ELISA Kit, R&D, M6000B, USA; Mouse IL-1 beta/IL-1F2 Quantikine ELISA Kit, R&D, MLB00C, USA; Mouse TNF-alpha Quantikine ELISA Kit, R&D, MTA00B, USA; Mouse IL-10 Quantikine ELISA Kit, R&D, M1000B, USA). The mice were anesthetized by intraperitoneal injection of 1% sodium pentobarbital (10 ml/kg). After stimulating the hind paws of the mice without any response, the operation was performed, and L3-L5 spinal cords were obtained. The tissue was lysed with RIPA Lysis Buffer (C1053, Applygen, China). The supernatant was collected for cytokine detection. The medium of the N2A cell line and BV2 cell line was collected, and the supernatant was collected by centrifugation at 3000 rpm for 10 min. Experiments using the double-antibody sandwich method. The entire experimental process is completely in accordance with the experimental steps in the kit.

### Western blotting

The changes in IRG1, Nrf2, IL-10Rα, IL-10, P-STAT3, STAT3, and *β*-endorphin protein content were detected with western blotting. *β*-actin was used as the internal control. The mice were anesthetized by intraperitoneal injection of 1% sodium pentobarbital (10 ml/kg). After stimulating the hind paws of the mice without any response, the L3-L5 spinal cords were obtained. The tissue was lysed with RIPA Lysis Buffer. Then use the BCA protein determination kit (23227, Thermo Scientific, U.S.A.) for protein quantification. The protein samples were separated on a 10% SDS-PAGE gel (ZD304, ZOMANBIO, China), transferred to a PVDF membrane (1620177, Bio-rad, USA), sealed with 5% skimmed milk powder for 60 minutes, and then incubated with the primary antibodies at 4°C overnight: IRG1(1:500, 17805S, Cell Signaling Technology, USA), Nrf2 (1:1000, ab62352, Abcam, USA), IL-10Rα (1:1000, sc-28371, Santa Cruz, USA), IL-10 (1:1000, ab9969, Abcam, USA), P-STAT3 (1:1000, 9145T, Cell Signaling Technology, USA), STAT3 (1:1000, ab76315, Abcam, USA), β-endorphin (1:1000, ab10339, Abcam, USA), or β-actin (1:1000, sc-47778, Santa Cruz, USA). The next day, the membrane was washed and incubated with secondary antibodies (1:1000, Anti-rabbit IgG: 7074S, Anti-mouse IgG: 7076S, Cell Signaling Technology, USA) or 1 hour at room temperature. Finally, the protein was detected by an ECL reagent (WBKLS0500, Millipore, USA). The relative intensity of the protein is quantified by ImageJ software.

### Immunofluorescent staining

Fourteen days after surgery, mice were anesthetized with 1% sodium pentobarbital (10 ml/kg) and perfused *via* the heart with 0.01 M PBS and 4% paraformaldehyde. The L3-L5 spinal cord was removed in 4% paraformaldehyde overnight at 4°C. The tissues were dehydrated in gradients using 20% and 30% sucrose solutions. After embedding the tissue, frozen sections (16μm) were performed using a cryomicrotome (CM3050 S, Leica Microsystems, Germany). Tissue sections were subjected to antigen repair using sodium citrate and treated at 95°C for 10 min, followed by the following treatments: The samples were treated with 1% Triton-X-100 for 15 min, washed three times with 0.01 M PBS for 5 min each, and incubated with 0.3% H2O2 for 10 min. The samples were washed three times with 0.01 M PBS for 5 min each. Blocked with 5% goat serum containing 0.3% Triton-X-100 for 30 min, sections were incubated overnight at 4°C with each of the following antibodies in 1% goat serum dissolved in 0.3% Triton-X-100: IL-10 (1:200, ab9969, Abcam, USA), NeuN (1:500; MAB377, Merck, USA). After washing with 0.01 M PBS 3 times, 5 minutes each time, the secondary antibody (1:500; Invitrogen, USA) was incubated for 1 h at room temperature. After washing, the sections were fixed with DAPI (AB104139, ABCAM, USA) in the dark and then covered with cover slides. Immunohistochemical images were taken using a microscope (DS-IR2, Nikon), and IL-10-expressing neurons in the ipsilateral spinal cord were counted using ImageJ software.

### Spinal dorsal horn recording

The CCI animals at 14th days were anesthetized with isoflurane and mechanically ventilated through a tracheotomy tube. The lumbar enlargement of the spinal cord was exposed by a laminectomy between vertebral levels T12 and L1. The recording of wide dynamic range (WDR) neurons was through parylene-coated tungsten microelectrodes (impedance 1-3 MV; Frederick Haer Company, Brunswick, ME) placed at a depth of 300 to 500 μm from the dorsal surface of L4 spinal cord level. Only WDR neurons with defined receptive fields in the plantar region of the hind paw were studied. The analog signals were amplified, filtered, displayed on an oscilloscope, and collected with a real-time computer-based data acquisition and processing system (CED 1401; Cambridge Electronic Design, Cambridge, United Kingdom). The WDR neuronal responses were evoked by graded peripheral intracutaneous electrical stimulation (0.1-10 mA, 1 ms). A train of 0.5-Hz electrical stimulation (16 pulses, 0.2 ms, 1.5×C-component threshold) was used to induce a windup response.

### Statistical analysis

All data are expressed as mean ± SEM and analyzed using GraphPad Prism 8.0 (GraphPad Software, U.S.A). Use two-tailed unpaired student’s *t*-tests, one-way, two-way ANOVA tests, or paired student’s *t*-tests and Dunnett or Tukey’s multiple comparison test as needed to estimate statistical significance. A P value less than 0.05 is considered statistically significant.

## Results

### The level of IRG1/itaconate was increased in the spinal cord after CCI

**Figure 1:**
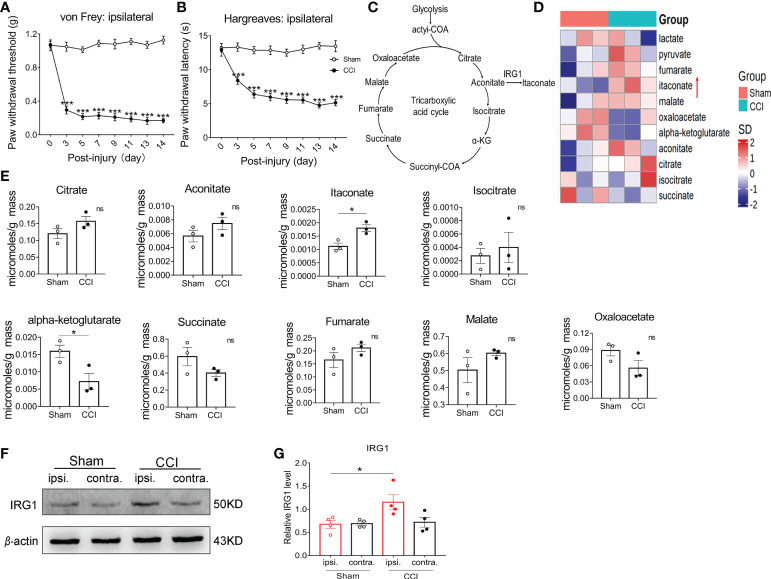
The content of itaconate and IRG1 increased in the spinal cord of CCI mice. The CCI model mice exhibited the significant decrease in the PWT **(A)** and PWL **(B)** of ipsilateral hind paw from 3rd day post nerve injury. Data are presented as mean ± SEM. ***p<0.001 vs. Sham, by two-way ANOVA with Tukey’s multiple comparisons test (n = 10). **(C)** Diagram of the tricarboxylic acid (TCA) cycle. **(D)** The heat map of the mass spectrometry result showed that the content of the TCA cycle intermediates in the L3-L5 segment of the ipsilateral spinal cord from sham and CCI mice at 14th days post nerve injury. **(E)** The changes in the content of the intermediate products of the TCA cycle. Data are presented as mean ± SEM. *p < 0.05 vs. Sham, by Paired t tests (n = 3). **(F)** The representative image of western blot for the expression of IRG1 in the L3-L5 segments of ipsilateral and contralateral spinal cord from sham and CCI mice. **(G)** The level of IRG1 protein in the ipsilateral spinal cord at 14th days after nerve injury significantly increased. Data are presented as mean ± SEM. *p<0.05 vs. Sham, followed by One-way ANOVA with Dunnett’s multiple comparisons test (n = 4). ns, no significance.

We first established the chronic constriction nerve injury (CCI) model in male C57BL/6 (wildtype, WT) mice. Compared with the preinjury baseline, the paw withdrawal threshold (PWT) and paw withdrawal latency (PWL) of the nerve-injured (ipsilateral) hind paw to mechanical and heat stimuli were significantly decreased at 3 days and achieved peak levels 7-9 days post-CCI (**Figures 1A, B**). To explore the changes in the content of itaconate in the spinal cord after peripheral nerve injury, the ipsilateral L3-L5 segments of the spinal cord were collected from CCI and sham-operated mice to conduct the mass spectrometry on day 14 post nerve injury. Among the intermediates of the TCA cycle (**Figure 1C**), the content of spinal itaconate increased and α-ketoglutarate decreased respectively post-CCI (**Figures 1D, E**). Consistent with the upregulation of itaconate, the protein level of IRG1 was elevated in the ipsilateral L3-L5 segments of the spinal cord on day 14 post-CCI (**Figures 1F, G**). These results suggested that the spinal endogenous IRG1/itaconate may be involved in the development of pain hypersensitivity in the CCI model.

### Endogenous IRG1/itaconate played an analgesic effect in the CCI model

**Figure 2:**
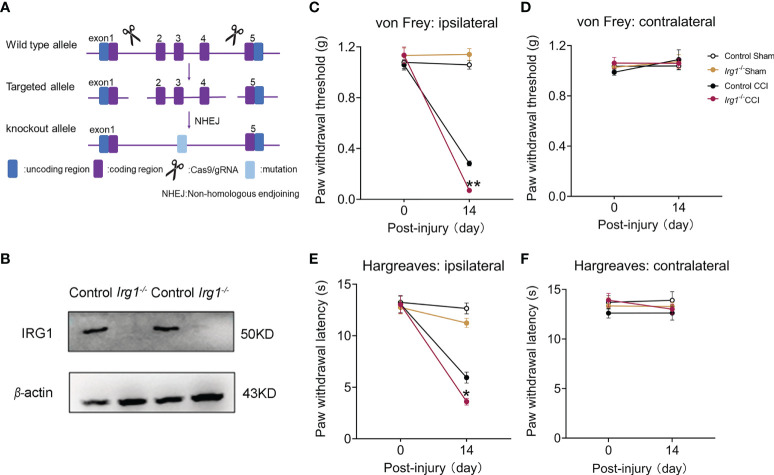
The deficiency of IRG1 aggravated the pain hypersensitivity after nerve injury. **(A)** Schematic diagram of constructing *Irg1* ^-/-^ mice. **(B)** At 14th days post injury, the protein level of IRG1 in L3-L5 segments of the ipsilateral spinal cords from control CCI and *Irg1* ^-/-^ CCI mice. **(C, D)** At 14th days post injury, the PWT of ipsilateral and contralateral hind paw to von Frey test in Sham and CCI mice. **(E, F)** At 14th days post injury, the PWL of ipsilateral and contralateral hind paw to Hargreaves test in Sham and CCI mice. Data are presented as mean ± SEM. **p<0.01, *p<0.05 vs. Control CCI, followed by two-way ANOVA with Tukey’s multiple comparisons test (n = 10).

To examine the direct correlation between endogenous IRG1/itaconate and pain behaviors in the CCI model, we constructed global *Irg1* ^-/-^ mice with CRISPR/Cas9 technology (**Figure 2A**). By using control and *Irg1* ^-/-^ mice to establish the CCI model, we verified that the IRG1 protein was completely depleted in the L3-L5 spinal cord in *Irg1* ^-/-^ CCI mice with western blotting (**Figure 2B**). On the 14^th^ day post-injury, *Irg1* ^-/-^ CCI mice exhibited lower PWT of the ipsilateral hind paw compared with those in control CCI mice (**Figure 2C**). The PWT of the contralateral hind paw of *Irg1* ^-/-^ CCI mice did not show a significant difference between the control CCI and sham animals (**Figure 2D**). The *Irg1* ^-/-^ CCI mice also exhibited lower PWL of the ipsilateral hind paw (**Figure 2E**) and similar PWL of the contralateral hind paw (**Figure 2F**) compared with those in control CCI mice. These findings suggested that the loss of endogenous IRG1/itaconate aggravated both mechanical allodynia and heat hyperalgesia in nerve-injured mice.

### Systemic and local application of 4-octyl itaconate (4-OI) attenuated mechanical and thermal hypersensitivity in the CCI mice

**Figure 3:**
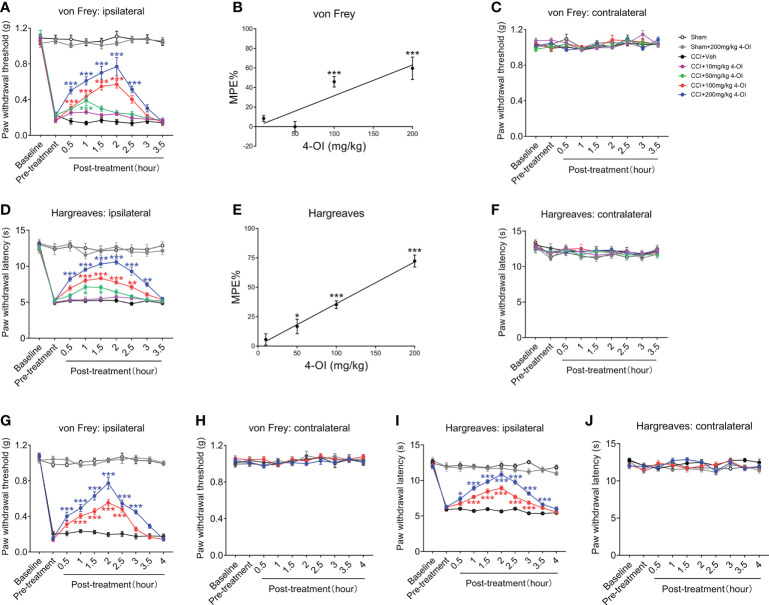
Intraperitoneal injection of 4-OI suppressed the neuropathic pain of the both sexes CCI mice. **(A)** The effect of systemic application of 4-OI on the PWT of ipsilateral hind paw in male CCI mice at 14th days post injury. **(B)** The maximum possible effect (MPE) of PWT at 1 hour post 4-OI was calculated. **(C)** The effect of 4-OI on the PWT of contralateral hind paw. **(D)** The effect of systemic application of 4-OI on the PWL of ipsilateral hind paw in male CCI mice at 14th days post injury. **(E)** The MPE of PWL at 1 hour post 4-OI was calculated. **(F)** The effect of 4-OI on the PWL of contralateral hind paw. **(G)** The effect of systemic application of 4-OI on the PWT of the ipsilateral hind paw in female CCI mice at 14th days post-injury. **(H)** The effect of 4-OI on the PWT of the contralateral hind paw. **(I)** The effect of systemic application of 4-OI on the PWL of the ipsilateral hind paw in female CCI mice at 14th days post-injury. **(J)** The effect of 4-OI on the PWL of the contralateral hind paw. Data are presented as mean ± SEM. ***p<0.001, **p<0.01, *p<0.05 vs. CCI + Veh, followed by two-way ANOVA **(A, D, G, I)** and one-way ANOVA **(B, E)** with Tukey’s and Dunnett’s multiple comparisons test (n = 12).

Next, we explored the effect of exogenous itaconate on the mechanical allodynia and heat hyperalgesia of the CCI mice. Since itaconate is difficult to penetrate the cell membrane and is not stable in *in vivo* conditions (31, 32), we elevated the level of itaconate by application of 4-OI, a derivative of itaconate, which has better cell permeability and transforms to itaconate through esterlysis (33–35). We first examined the effects of systemic administration of 4-OI with various doses on the mechanical and thermal hypersensitivity in male CCI mice. Compared with the preinjury baseline, the PWT (**Figure 3A**) of the ipsilateral hind paw to mechanical and heat stimuli were significantly decreased at 14^th^ days post-CCI. We intraperitoneally injected various doses of 4-OI (10, 50, 100, 200 mg/kg) to the CCI mice on day 14, and then detected the PWT and PWL at different time points. The results showed that only 10 mg/kg 4-OI didn’t affect the PWT (**Figure 3A**) of ipsilateral hind paws of CCI mice at all time points. The other three higher doses of 4-OI significantly increased the PWT (**Figure 3A**) of ipsilateral hind paws at different time points post-treatment. The MPE at 2 hours post-treatment indicated that the analgesic effect of 4-OI exhibited a dose-dependent manner (**Figure 3B**). The intraperitoneal application of 4-OI had no obvious effect on the PWT (**Figure 3C**) of contralateral hind paws of sham and CCI mice. Similar with the results of PWT, the intraperitoneal application of 4-OI dose-dependently increased the PWL of ipsilateral hind paw of CCI mice at different time points after treatment (**Figures 3D, E**), but did not change the PWL of contralateral hind paw (**Figure 3F**).

To explore whether 4-OI could induce an analgesic effect in both genders, we used female WT mice to establish the CCI model and applied the 4-OI by intraperitoneal injection. Administration of 100 and 200 mg/kg 4-OI significantly increased the PWT (**Figure 3G**) of the ipsilateral hind paws of the female CCI mice. The higher dose of 4-OI induced the analgesic effect at an earlier time point (0.5 hours post-treatment) and the effect was maintained longer (3-3.5 hours post-treatment) (**Figure 3G**). The application of 4-OI did not affect the PWT (**Figure 3H**) of the contralateral hind paws of the female sham-operated and CCI mice. The 100 and 200 mg/kg 4-OI also significantly increased the PWL of the ipsilateral hind paws of the female CCI mice (**Figure 3I**), but did not affect the PWL of the contralateral hind paw (**Figure 3J**). These results suggested that 4-OI alleviated neuropathic pain, with no sex differences.

**Figure 4:**
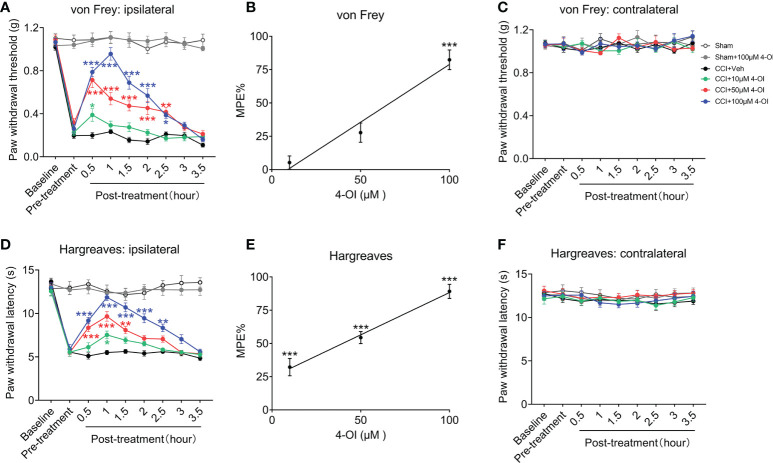
Intrathecal injection of 4-OI alleviated the pain hypersensitivity in male CCI mice. **(A)** The effect of local application of 4-OI on the PWT of ipsilateral hind paw in male CCI mice at 14th days post injury. **(B)** The maximum possible effect (MPE) of PWT at 2 hours post 4-OI was calculated. **(C)** The effect of 4-OI on the PWT of contralateral hind paw. **(D)** The effect of local application of 4-OI on the PWL of ipsilateral hind paw in male CCI mice at 14th days post injury. **(E)** The MPE of PWL at 2 hours post 4-OI was calculated. **(F)** The effect of 4-OI on the PWL of contralateral hind paw. Data are presented as mean ± SEM. ***p<0.001, **p<0.01, *p<0.05 vs. CCI + Veh, followed by two-way ANOVA **(A, D)** and one-way ANOVA **(B, E)** with Tukey’s and Dunnett’s multiple comparisons test (n = 12).

In order to detect the local effects of 4-OI, we conducted intrathecal injection of 4-OI with different concentrations in another group of male CCI mice. The results also showed that intrathecal injection of different doses of 4-OI could alleviate the mechanical allodynia of male CCI mice at different time points post-treatment (**Figure 4A**). In addition, the MPE at 1 hour post-treatment showed that the analgesic effect of the intrathecal application of 4-OI was dose-dependently (**Figure 4B**). The PWT of the contralateral hind paws of Sham-operated and CCI mice were unchanged after intrathecal application of 4-OI (**Figure 4C**). The intrathecal injection of 4-OI also dose-dependently attenuated the thermal hyperalgesia of male CCI mice at 14^th^ days post injury (**Figures 4D, E**). The PWL of the contralateral hind paws was unaffected by intrathecal application of 4-OI (**Figure 4F**). Our experimental results proved that 4-OI treatment was able to attenuate the mechanical allodynia and heat hyperalgesia of male CCI mice. The potential active site of 4-OI could be at the spinal cord level.

### 4-OI inhibited the C-component and windup responses of WDR neurons in CCI mice

**Figure 5:**
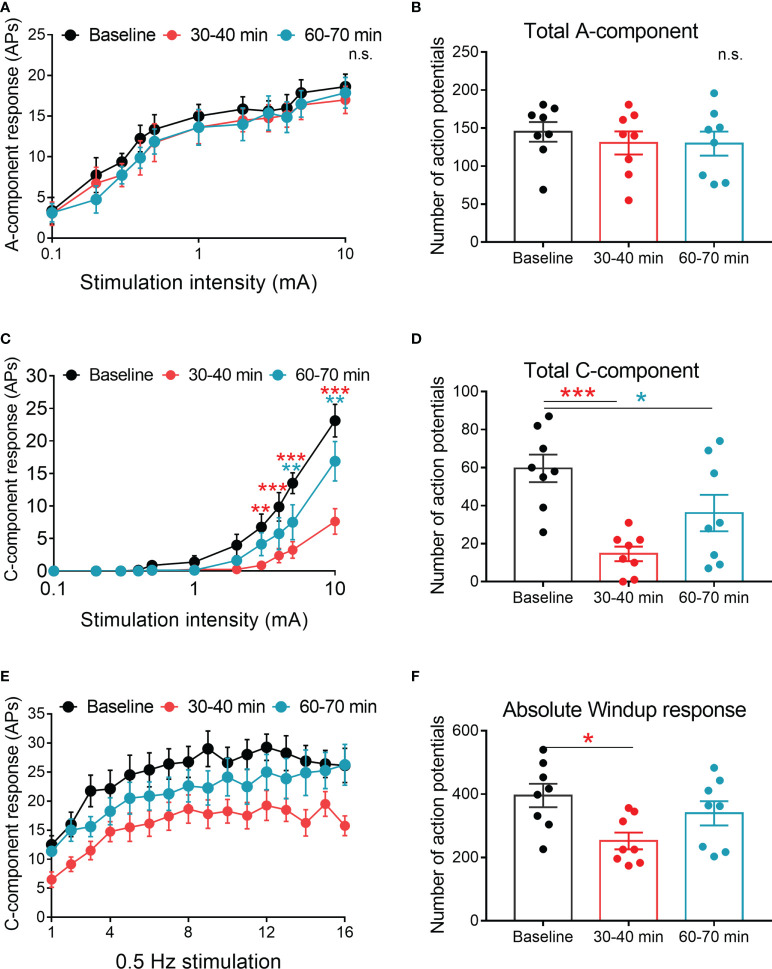
Spinal 4-OI attenuated the C-component and windup responses of WDR neurons in CCI mice. **(A)** The stimulus-response functions of A-component to graded electrical stimulation before, 30-40 and 60-70 minutes after spinal 4-OI (100 μM, 10 μl). **(B)** The total number of action potentials (APs) in the A-component produced in response to graded electrical stimuli. **(C)** The stimulus-response functions of C-component before, 30-40 and 60-70 minutes after 4-OI. **(D)** The total APs of C-component produced in response to graded electrical stimuli. **(E)** Windup function in WDR neurons was examined before, 30-40 and 60-70 minutes after 4-OI. **(F)** The total number of C-components evoked by windup-inducing stimulation after 4-OI. n = 8/group; Data are presented as mean ± SEM. *p < 0.05, **p < 0.01, ***p < 0.001 vs baseline by two-way ANOVA with Tukey’s multiple comparisons test **(A, C, E)** and paired t-test **(B, D, F)**. n.s., no significance.

Next, we investigated whether 4-OI affects the evoked response of spinal WDR neurons in CCI mice. According to different response latencies produced by intracutaneous electrical stimulation

of the hind paw, the A (0-50 ms)- and C (50-250 ms)-components of WDR neuronal responses can be distinguished. At 30-40 and 60-70 mins after spinal application of 4-OI (100 μM, 10 μl), 4-OI didn’t affect the response of A-component evoked by graded electrical stimuli with increasing amplitudes (**Figures 5A, B**). However, the C-component responses to 3-, 4-, 5-, and 10-mA electrical stimuli at 30-40 mins post 4-OI, as well as the total number of action potentials in the C-component, were also significantly decreased at 30-40 and 60-70 mins after 4-OI application (**Figures 5C, D**). Repetitive electrical stimulation (0.5 Hz) with 1.5 times the C-fiber threshold induced windup in the C-component of the WDR neuronal response, which represents the short-term sensitization of WDR neurons. Notably, at 30-40 mins after local administration of 4-OI (100 μM, 10 μl), both the windup function and total windup response were attenuated from those observed before drug treatment (**Figures 5E, F**).

### The effect of 4-OI on the release of inflammatory and anti-inflammatory factors

**Figure 6:**
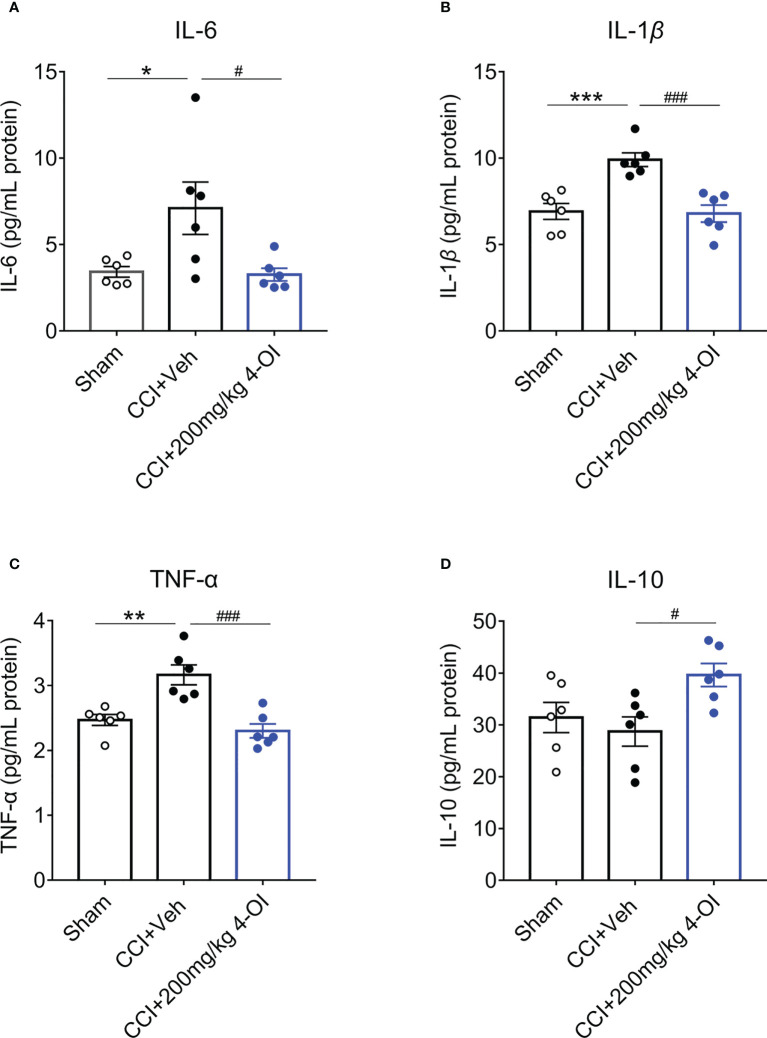
4-OI affected the spinal content of cytokine in CCI model. The expressions of inflammatory mediators IL-6 **(A)**, TNF-α **(B)** and IL-1β **(C)** significantly increased in the ipsilateral spinal cord of CCI group at 14th days post nerve injury. At 2 hours post systemic injection of 200 mg/kg 4-OI, the expressions of IL-6 **(A)**, TNF-α **(B)** and IL-1β **(C)** were markedly downregulated as well as the expression of IL-10 was upregulated **(D)**. Data are presented as mean ± SEM. ***p<0.001, **p<0.01, *p<0.05 vs. Sham, ^###^ *p*<0.001, ^#^ *p*<0.05 vs. CCI + Veh, followed by One-way ANOVA with Dunnett’s multiple comparisons test (n = 6).

Since itaconate has been suggested as the inflammatory regulator, we speculated that the analgesic effect of IRG1/itaconate might through the immunoregulatory effect. Therefore, we examined the changes of some classical cytokine expressions in the L3-L5 spinal cord segments from the male CCI mice after intraperitoneal administration of vehicle or 4-OI. According to the results of ELISA experiments, the spinal protein content of interleukin (IL)-6, Tumor necrosis factor (TNF)-α, and IL-1β was significantly decreased at 2 hours post 200 mg/kg 4-OI injection (**Figures 6A–C**), while the expression of anti-inflammatory factor IL-10 was significantly increased (**Figure 6D**). As the IL-10 was known to inhibit pain hypersensitivity, those results suggested that 4-OI might upregulate the IL-10 pathway to suppress the pain behaviors in the CCI model.

### 4-OI activated the IL-10/STAT3 pathway and promoted the release of β-endorphin

**Figure 7:**
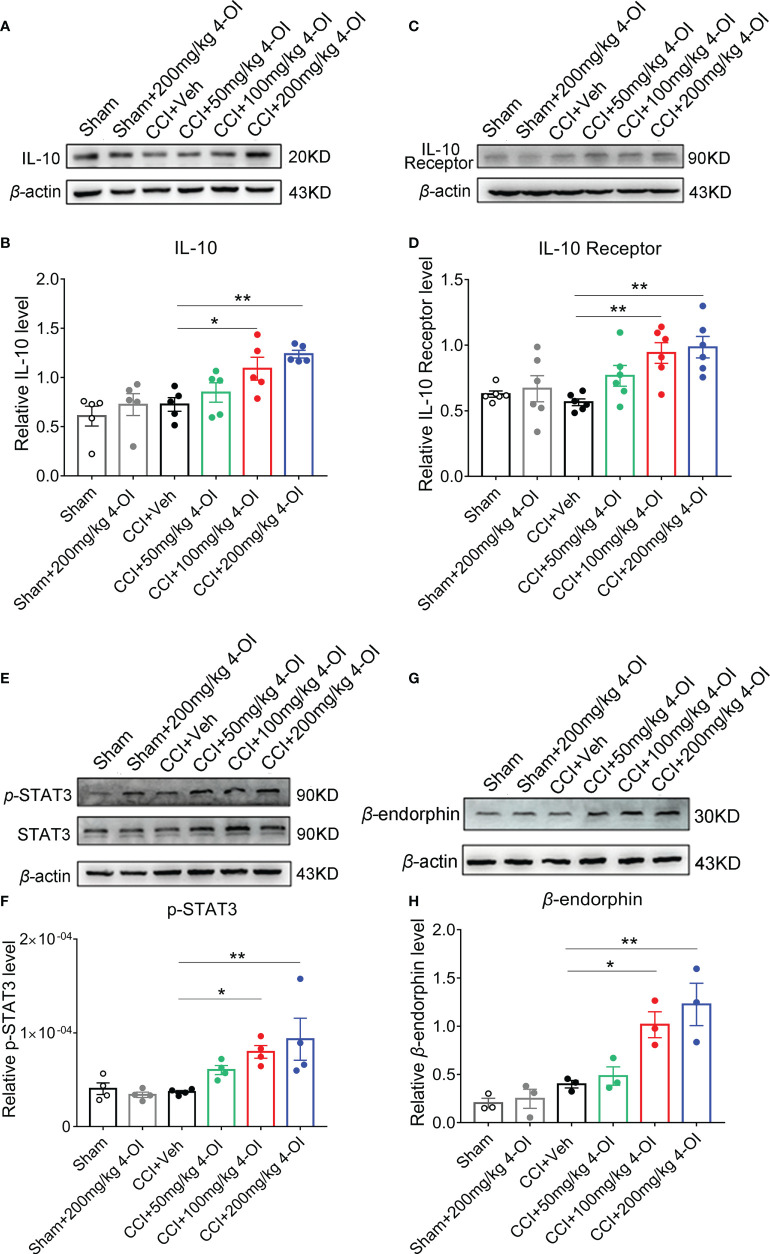
4-OI activated the IL-10/β-endorphin pathway. At 14th days post injury, different doses of 4-OI were applied to CCI mice by intraperitoneal injection. Compared with the vehicle group, the expressions of IL-10 **(A, B)**, IL-10 receptor **(C, D)**, p-STAT3 **(E, F)** and β-endorphin **(G, H)** in ipsilateral spinal cord of CCI mice were elevated at 2 hours post 100 and 200 mg/kg 4-OI application. Data are presented as mean ± SEM. **p<0.01, *p<0.05 vs. CCI + Veh, followed by One-way ANOVA with Dunnett’s multiple comparisons test (n = 3-6).

We next detected the protein level of the IL-10 pathway in the L3-L5 segments of the ipsilateral spinal cord from male CCI mice at 2 hours post-4-OI treatment. The expression of IL-10 (**Figures 7A, B**) and IL-10 receptor (**Figures 7C, D**) significantly increased after 100 and 200 mg/kg 4-OI treatment. IL-10 binds to the IL-10 receptor and activates the downstream signal transducer and activator of transcription 3 (STAT3) signal. The phosphorylation of STAT3 (p-STAT3) leads to the increase of nuclear translocation of STAT3, which enhances the transcription of its target genes. After treatment of 100 and 200 mg/kg 4-OI, the level p-STAT3 (**Figures 7E, F**) and the downstream β-endorphin (**Figures 7G, H**) significantly increased. As the β-endorphin was a well-known endogenous analgesia, our findings indicated that the application of 4-OI might suppress neuropathic pain through the IL-10/STAT3/β-endorphin pathway.

### The deficiency of *IL-10* disturbed the analgesia of 4-OI in the CCI model

**Figure 8:**
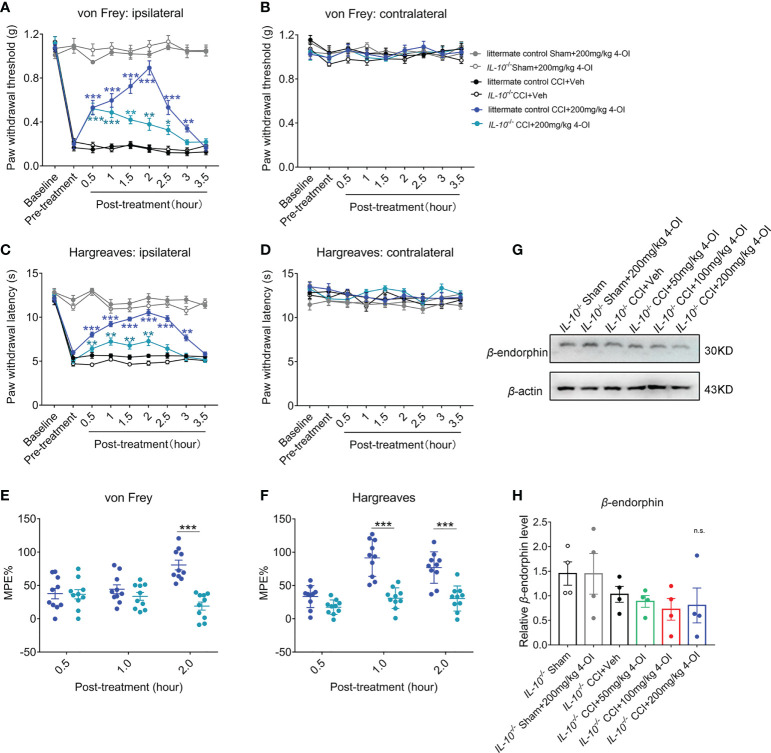
The deficiency of IL-10 disturbed the analgesia of 4-OI in neuropathic pain. **(A)** The PWT of ipsilateral hind paw was elevated after 200 mg/kg 4-OI in *IL-10* ^-/-^ and littermate control CCI mice. **(B)** The PWT of contralateral hind paw was unchanged by 4-OI treatment. **(C)** The PWL of ipsilateral hind paw was increased after 200 mg/kg 4-OI in *IL-10* ^-/-^ and littermate control CCI mice. **(D)** The PWL of contralateral hind paw was unchanged by 4-OI treatment. Data are presented as mean ± SEM. ***p<0.001, **p<0.01, *p<0.05 vs. CCI + Veh followed by two-way ANOVA with Tukey’s multiple comparisons test (n = 10) **(A, C)**. **(E)** The MPE of 4-OI was calculated for mechanical allodynia at 0.5 and 2 hours post injection. **(F)** The MPE of 4-OI was calculated for heat hyperalgesia at 1 and 2 hours post injection. Data are presented as mean ± SEM. ***P < 0.001 vs. littermate control CCI+4-OI 200 mg/kg followed by two-way ANOVA with Tukey’s multiple comparisons test (n = 10) **(E, F)**. **(G)** The representative image of western blot for the expression of β-endorphin at 2 hours post 4-OI treatment in *IL-10* ^-/-^ sham and *IL-10* ^-/-^ CCI group. **(H)** The spinal level of β-endorphin was unchanged after different doses of 4-OI. Data are presented as mean ± SEM, n.s. vs. CCI + vehicle followed by one-way ANOVA with Dunnett’s multiple comparisons test (n = 4). n.s., no significance.

To verify the role of IL-10 in the analgesia of 4-OI, we used male *IL-10* ^-/-^ mice to establish the CCI model. At 14 days post-nerve injury, we applied 200 mg/kg 4-OI by intraperitoneal injection to the CCI and sham-operated mice in *IL-10* ^-/-^ and littermates. Consistent with our previous results, 200 mg/kg 4-OI induced a significant increase of PWT of the ipsilateral hind paws of littermate control CCI mice from 0.5 to 3 hours post-treatment (**Figure 8A**). The PWT of the contralateral hind paws were unaffected by 4-OI administration (**Figure 8B**). Similar with the results of the PWT, 200 mg/kg 4-OI also induced a significant increase of PWL of the ipsilateral hind paws (**Figure 8C**) and did not affect the PWL of the contralateral hind paws (**Figure 8D**) of littermate control CCI mice. In *IL-10* ^-/-^ CCI mice, although 200 mg/kg 4-OI still alleviated the mechanical allodynia and heat hyperalgesia of ipsilateral hind paws, the results of MPE indicated that analgesic effects of 4-OI on mechanical (**Figure 8E**) and heat hypersensitivity (**Figure 8F**) was markedly weaker in *IL-10* ^-/-^ CCI group compared with that in littermate control CCI group. Moreover, we found that the spinal protein level of β-endorphin was not upregulated at 2 hours after 4-OI treatment in *IL-10* ^-/-^ CCI mice (**Figures 8G, H**). These results suggested that the IL-10/β-endorphin pathway greatly contributed to the analgesic effect of 4-OI treatment on neuropathic pain.

### Itaconate induced the upregulation of neuronal IL-10 by the Nrf2 pathway

**Figure 9:**
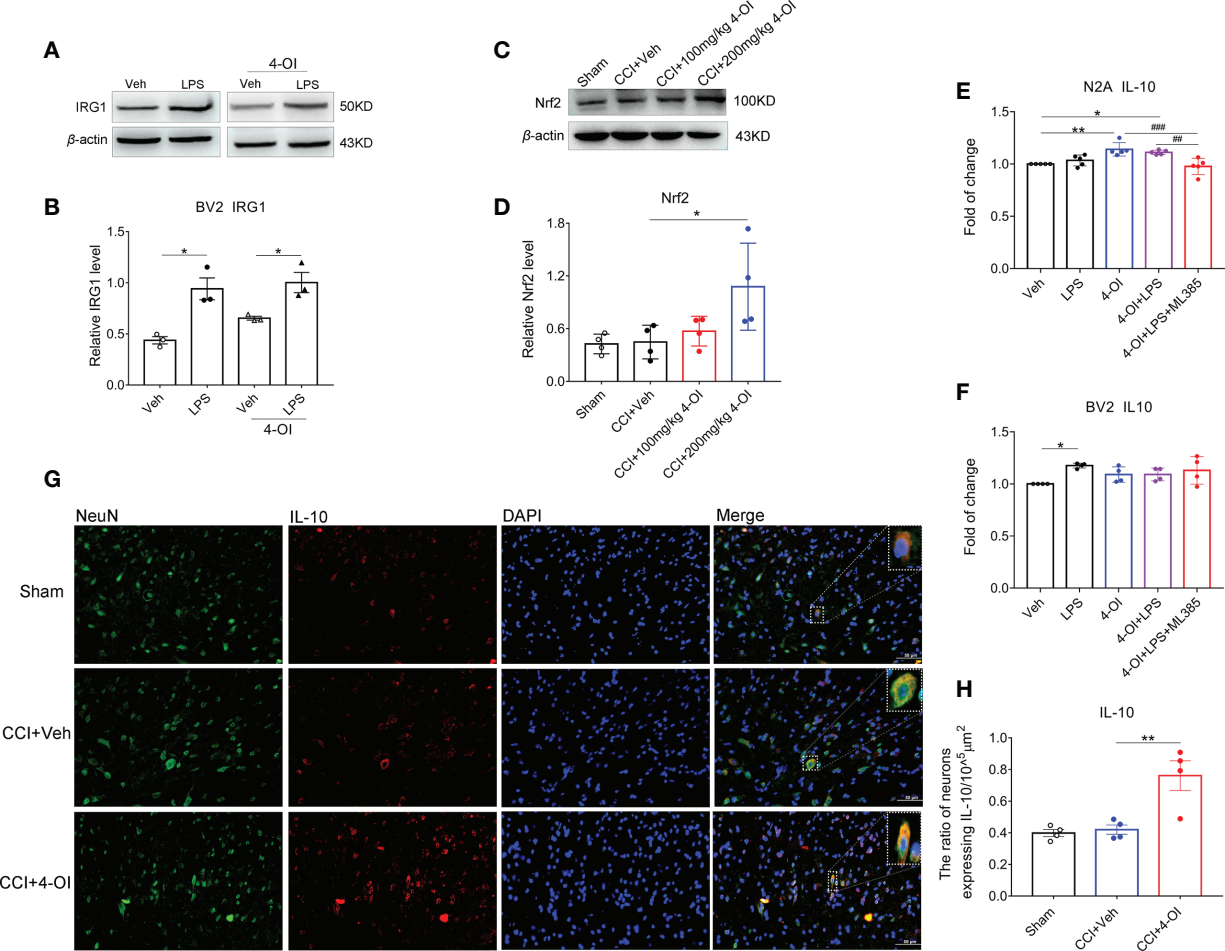
Itaconate promoted the neuronal expression of IL-10 by activation of Nrf2. The representative image **(A)** and statistical analysis **(B)** of IRG1 protein level in BV2 cells under LPS and 4-OI treatment. Data are presented as mean ± SEM. *p<0.05 vs. Veh followed by One-way ANOVA with Dunnett’s multiple comparisons test (n = 3). The representative image **(C)** and statistical analysis **(D)** of Nrf2 protein level in the ipsilateral spinal cord at 2 h after the intraperitoneal injection of 4-OI in male CCI mice at 14^th^-day post-injury. Data are presented as mean ± SEM. *p<0.05 vs. CCI + Veh followed by One-way ANOVA with Dunnett’s multiple comparisons test (n = 4). The release of IL-10 after the treatment of 4-OI, LPS, and ML385 in the culture medium of N2A cells **(E)** and BV2 cells **(F)** was detected by ELISA. **p<0.01, *p<0.05 vs. Veh, ###*p*<0.001, ##*p*<0.01 vs. 4-OI + LPS + ML385 followed by One-way ANOVA with Dunnett’s multiple comparisons test (n = 4-5). **(G)** The colocalization of neuronal marker NeuN and IL-10 in the spinal dorsal horn of male CCI mice. **(H)** Statistical analysis of the proportion of spinal neurons expressing IL-10. Data are presented as mean ± SEM. **p<0.01 vs. CCI + Veh followed by One-way ANOVA with Dunnett’s multiple comparisons test (n = 4).

As previous study suggested the itaconate could be produced in macrophages, we examined whether itaconate also expressed in microglia and exhibited the response to noxious stimulus. Under the stimulation of LPS, the protein level of IRG1 was upregulated in BV2 cells with or without 4-OI treatment (**Figures 9A, B**), which suggested the endogenous itaconate might be produced by spinal microglia in CCI model. To explore whether the regulation of IL-10 by itaconate depended on the Nrf2 pathway, we first examined the expression of Nrf2 in the ipsilateral spinal cord of male CCI mice with 4-OI injection. The results indicated that the protein level of spinal Nrf2 was increased at 2 h post intraperitoneal injection of 200 mg/kg 4-OI (**Figures 9C, D**). Since both the neurons (36, 37) and microglia (38) could produce the IL-10, then we used the N2A cells and BV2 cells to mimic neurons and microglia respectively. We found that after the treatment of 4-OI and 4-OI+LPS, the IL-10 released from the N2A cells was increased compared to that in the vehicle group (**Figure 9E**). More importantly, the increase of IL-10 was inhibited by Nrf2 inhibitor ML385 (**Figure 9E**). However, the release of IL-10 was not affected by 4-OI treatment in BV2 cells (**Figure 9F**). Finally, by double immunofluorescent staining, we proved that the proportion of spinal neurons expressing IL-10 was increased after 4-OI treatment in male CCI mice (**Figures 9G, H**). These results suggested that the increased IL-10 was mainly secreted from spinal neurons through the Nrf2 pathway.

## Discussion

Although the accumulation of itaconate can be triggered by Lipopolysaccharide (LPS) stimulation in macrophages (18), it is unknown whether the content of itaconate in nervous system is altered after the nerve injury and how it affects pain behaviors. In our current manuscript, we first showed that the level of immune response gene 1 (IRG1)/itaconate in the spinal cord of the L3-L5 segment was up-regulated post peripheral sciatic nerve injury. By using the *Irg1* ^-/-^ mice, we found that the depletion of *Irg1* aggravated the pain hypersensitivity induced by nerve injury. In addition, we proved that the pain hypersensitivity of WT CCI mice was mitigated by systemic or local application of 4-octyl itaconate (4-OI), in which analgesic effect was dose-dependent in male and female mice. Finally, we demonstrated that 4-OI produced an analgesic effect partially by up-regulating the interleukin (IL)-10/STAT3/β-endorphin pathway. Our research suggested that IRG1/itaconate could be potential drug target for the treatment of neuropathic pain.

There was no report to study the content of endogenous itaconate in the spinal cord and its relationship to peripheral nerve injury until this manuscript. By using the liquid chromatography-mass spectrometry (LC-MS) technology, we found the basal level of itaconate was very low in the L3-L5 segment of the spinal cord, but it is the only metabolite of tricarboxylic acid (TCA) cycle significantly increased as the pain hypersensitivity exhibited. The production of itaconate represents metabolic remodeling in the body and disturbs the normal TCA cycle (13, 39–41). Since itaconate has been suggested to exhibit anti-inflammatory function (13), our findings indicated that the cells located in the spinal cord spontaneously produced anti-inflammatory substances under pathological conditions to resist the damage caused by inflammation to the body.

The content level of itaconate is most likely dependent on the IRG1 protein level/activity, which works as an enzyme catalyzing the production of itaconate by decarboxylating *cis*-aconitate (42, 43). In line with the increase of itaconate, the expression of IRG1 protein increased in the ipsilateral spinal cord of CCI mice compared with that in the spinal cord of sham-operated animals or contralateral side of the CCI mice. To investigate the function of endogenous itaconate in neuropathic pain, we used global *Irg1* ^-/-^ mice to establish the CCI model and found the pain behaviors were more remarkable at 14 days post-injury (dpi) in the *Irg1* ^-/-^ CCI mice than those in control CCI mice. This result supported our hypothesis that IRG1/itaconate played a beneficial effect as endogenous analgesia after peripheral nerve injury. Nevertheless, although the content of itaconate was statistically increased from the physiological conditions, it was still too low to revert the pain hypersensitivity, which meant the exogenous itaconate could be a potential analgesia.

Since itaconate is easily metabolized and is decomposed into pyruvate and acetyl-CoA before entering the cell under *in vivo* conditions (31, 32), the itaconate derivatives are usually used to elevate the content of itaconate in the body. The 4-OI is a cell-permeable itaconate derivative, which can reduce the production of cytokines and protect the body from LPS-induced lethality (33–35). We first conducted the systemic injection of 4-OI at various doses to test its analgesic effect. Except for the lowest dose (10 mg/kg), the three higher doses (50, 100, 200 mg/kg) of 4-OI suppressed the mechanical and heat hypersensitivity in a dose-dependent manner in the CCI model. To further confirm the active site of 4-OI, we locally applied it by intrathecal injection and found a similar analgesic effect at higher concentrations (50, 100 μM) of 4-OI. Meanwhile, the effective time of treatment was earlier by intrathecal application, which meant the potential target of 4-OI should be at the spinal cord level. We also proved that 4-OI alleviated the hyperalgesia in female CCI mice, which suggested the itaconate could be a potential painkiller for both genders.

As the production of itaconate is the result of the metabolic remodeling of the TCA cycle (42, 44), the application of the high dose of itaconate may affect the energy production in the TCA cycle (17, 31, 45, 46). However, there is no relevant report on whether the treatment of itaconate induces any side effects. In our study, we observed that the mice appeared quiet and unwilling to move after the systemic administration of 200 mg/kg 4-OI. The phenomenon might be a result of the reduction of energy consumption and lasted less than one hour. Other doses of 4-OI with systemic or local application did not induce an obvious impact on the behaviors of mice. These results suggested that the side effects of 4-OI should be completely evaluated, especially in the treatment of central nervous system disorders.

Macrophages also secrete large amounts of IL-10 through interferon (IFN)-β pathway other than itaconate. The IL-10 is released to the extracellular and then binds to its receptor IL-10R on the macrophages. One study shows that IL-10/IL-10R leads to the decrease of IRG1 protein and itaconate production (29). However, there is a lack of reports on whether itaconate could regulate the production of IL-10. In our experiments, we found that systemic application of 4-OI at 100 and 200 mg/kg promoted the expression of IL-10 and activated the downstream pathway in the spinal cord of the CCI mice. To verify the role of IL-10 in the analgesic effect of 4-OI, we used *IL-10* ^-/-^ mice to establish the CCI model. Compared with the results in control CCI mice, the analgesic effect of 200 mg/kg 4-OI was disturbed in the *IL-10* ^-/-^ CCI mice. The spinal level of β-endorphin post-4-OI treatment was also unchanged in *IL-10* ^-/-^ CCI mice, which was significantly elevated in WT CCI mice. Since the activation of the IL-10/β-endorphin pathway can alleviate neuropathic pain (47, 48), our results suggested that exogenous 4-OI could induce the analgesia by promoting the spinal level of IL-10/β-endorphin in neuropathic pain. It should be noted that 200 mg/kg 4-OI still exhibited a weak analgesic effect in *IL-10* ^-/-^ CCI mice, which indicated other analgesic mechanisms were involved in the effect of 4-OI and needed to be investigated in the future.

It has been suggested that IRG1/itaconate could be induced in myeloid cells and neurons upon exposure to LPS and virus infection (49, 50). Here, we showed that the IRG1 expression in BV2 cells was upregulated under LPS stimulation, which suggested the microglia *in vivo* might be the source of spinal itaconate in CCI model and contributed to the endogenous analgesia. Itaconate activates the Nrf2 pathway and plays the anti-inflammatory function (18). Meanwhile, the Nrf2 pathway could regulate the transcription of Heme oxygenase-1 (HO-1) and contribute to the production of IL-10 (51, 52). We found that the level of Nrf2 in ipsilateral spinal cord of CCI mice was increased after 4-OI treatment. Interestingly, the concentration of IL-10 in supernatant was elevated under 4-OI and LPS treatment in an Nrf2-dependented manner in N2A cells. Consistent with this, the IL-10 expression in ipsilateral spinal cord was increased in neurons after systemic injection of 4-OI in CCI mice. Although myeloid cells are the main source of IL-10, there are some studies indicating the IL-10 also can be produced in neurons (36, 37), neuronal cell line (53), and neural stem cells (54). So, it needs further investigation to explain why 4-OI fails to promote the expression of IL-10 in microglia and elucidate the specific regulation mechanisms of itaconate on neuronal IL-10 production.

## Conclusion

In our study, we found that spinal cord itaconate content and IRG1 expression protein level were significantly elevated after peripheral nerve injury. The deficiency of *Irg1* aggravated both mechanical allodynia and heat hyperalgesia in nerve-injured mice. Additionally, exogenous administration of itaconate derivate 4-OI reduces hyperalgesia through the Nrf2/IL-10/STAT3/β-endorphin axis. Our research provides new ideas and drug targets for the treatment of chronic pain.

## Data availability statement

The original contributions presented in the study are included in the article. Further inquiries can be directed to the corresponding authors.

## Ethics statement

The animal study was reviewed and approved by the Capital Medical University Animal Care and Use Committee.

## Author contributions

QS and TH did most of the experiments and contributed equally. FY, QL and QS designed the overall approach, coordinated the study, and drafted the manuscript. YZ, XW, JL, WC, and CW helped to complete the Elisa test and the data analysis. DL, WW, TL, YD, ZL, ML, and DS helped to complete the behavioral tests and the data analysis. ZX, LH, MP, YM, and LS helped to complete the WB experiment and the data analysis. QS, TH, PW, JZ, QL, and FY prepared the manuscript and figures. All authors read and approved the final manuscript.

## Funding

This work was supported by National Natural Science Foundation of China (grant No.81971037 to F. Yang, No.81873790 and 32070735 to Q. Li, U20A20391 to P. Wang) and the Beijing Natural Science Foundation Program and Scientific Research Key Program of Beijing Municipal Commission of Education (KZ202010025033 to Q. Li).

## Conflict of interest

The authors declare that the research was conducted in the absence of any commercial or financial relationships that could be construed as a potential conflict of interest.

## Publisher’s note

All claims expressed in this article are solely those of the authors and do not necessarily represent those of their affiliated organizations, or those of the publisher, the editors and the reviewers. Any product that may be evaluated in this article, or claim that may be made by its manufacturer, is not guaranteed or endorsed by the publisher.

## Abbreviations

IRG1, immune-responsive gene 1; TCA, tricarboxylic acid cycle; CCI, chronic constriction nerve injury; 4-OI, 4-octyl itaconate; IL-10, interleukin-10; IL-6, interleukin-6; IL-1β, interleukin-1β; TNF-α, tumor necrosis factor-α; STAT3, signal transducer and activator of transcription 3; p-STAT3, phosphorylation of STAT3; IFN, interferon; NLRP3, inhibitor of NLR family pyrin domain containing 3; Keap1, kelch like ECH associated protein 1; Nrf2, nuclear factor erythroid 2-related factor 2; LPS, lipopolysaccharide; LC-MS, liquid chromatography-mass spectrometry; ELISA, enzyme-linked immunosorbent assay; SPF, specific-pathogen free; MPE, maximum possible effect; PWT, paw withdrawal threshold; PWL, paw withdrawal latency. Dpi, days post-injury.

## References

[1] Colloca L Ludman T Bouhassira D Baron R Dickenson AH Yarnitsky D. Neuropathic pain. Nat Rev Dis Primers (2017) 3:17002. doi: 10.1038/nrdp.2017.2 28205574 PMC5371025

[2] Pineda-Farias JB Loeza-Alcocer E Nagarajan V Gold MS Sekula R. Mechanisms underlying the selective therapeutic efficacy of carbamazepine for attenuation of trigeminal nerve injury pain. J Neurosci (2021) 41(43):8991–9007. doi: 10.1523/JNEUROSCI.0547-21.2021 PMC8549540 34446571

[3] Pinho-Ribeiro FA Verri WA Jr. Chiu IM. Nociceptor sensory neuron-immune interactions in pain and inflammation. Trends Immunol (2017) 38(1):5–19. doi: 10.1016/j.it.2016.10.001 27793571 PMC5205568

[4] Scheuren PS Rosner J Curt A Hubli M. Pain-autonomic interaction: A surrogate marker of central sensitization. Eur J Pain (2020) 24(10):2015–26. doi: 10.1002/ejp.1645 32794307

[5] Backryd E Themistocleous A Larsson A Gordh T Rice AS Tesfaye S. HGF, CSF-1, CD40 and 11 other inflammation-related proteins are associated with pain in diabetic neuropathy: exploration and replication serum data from the pain in neuropathy study (PiNS). Pain (2021) 163(5):897–909. doi: 10.1097/j.pain.0000000000002451 PMC9009322 34433766

[6] Baral P Udit S Chiu IM. Pain and immunity: implications for host defence. Nat Rev Immunol (2019) 19(7):433–47. doi: 10.1038/s41577-019-0147-2 PMC6700742 30874629

[7] Zhang Y Ma S Ke X Yi Y Yu H Yu D. The mechanism of annexin A1 to modulate TRPV1 and nociception in dorsal root ganglion neurons. Cell Biosci (2021) 11(1):167. doi: 10.1186/s13578-021-00679-1 34446102 PMC8393810

[8] Lenert ME Avona A Garner KM Barron LR Burton MD. Sensory neurons, neuroimmunity, and pain modulation by sex hormones. Endocrinology (2021) 162(8):bqab109. doi: 10.1210/endocr/bqab109 34049389 PMC8237991

[9] Alvarez C Abdalla H Sulliman S Rojas P Wu YC Almarhoumi R. RvE1 impacts the gingival inflammatory infiltrate by inhibiting the T cell response in experimental periodontitis. Front Immunol (2021) 12:664756. doi: 10.3389/fimmu.2021.664756 34012448 PMC8126725

[10] Strelko CL Lu W Dufort FJ Seyfried TN Chiles TC Rabinowitz JD. Itaconic acid is a mammalian metabolite induced during macrophage activation. J Am Chem Soc (2011) 133(41):16386–9. doi: 10.1021/ja2070889 PMC3216473 21919507

[11] Michelucci A Cordes T Ghelfi J Pailot A Reiling N Goldmann O. Immune-responsive gene 1 protein links metabolism to immunity by catalyzing itaconic acid production. Proc Natl Acad Sci USA (2013) 110(19):7820–5. doi: 10.1073/pnas.1218599110 PMC3651434 23610393

[12] Luan HH Medzhitov R. Food fight: Role of itaconate and other metabolites in antimicrobial defense. Cell Metab (2016) 24(3):379–87. doi: 10.1016/j.cmet.2016.08.013 PMC5024735 27626199

[13] Hooftman A O'Neill LAJ. The immunomodulatory potential of the metabolite itaconate. Trends Immunol (2019) 40(8):687–98. doi: 10.1016/j.it.2019.05.007 31178405

[14] Bambouskova M Gorvel L Lampropoulou V Sergushichev A Loginicheva E Johnson K. Electrophilic properties of itaconate and derivatives regulate the IkappaBzeta-ATF3 inflammatory axis. Nature (2018) 556(7702):501–4. doi: 10.1038/s41586-018-0052-z PMC6037913 29670287

[15] Mills EL Kelly B Logan A Costa ASH Varma M Bryant CE. Succinate dehydrogenase supports metabolic repurposing of mitochondria to drive inflammatory macrophages. Cell (2016) 167(2):457–70.e13. doi: 10.1016/j.cell.2016.08.064 27667687 PMC5863951

[16] Lampropoulou V Sergushichev A Bambouskova M Nair S Vincent EE Loginicheva E. Itaconate links inhibition of succinate dehydrogenase with macrophage metabolic remodeling and regulation of inflammation. Cell Metab (2016) 24(1):158–66. doi: 10.1016/j.cmet.2016.06.004 PMC5108454 27374498

[17] Murphy MP O'Neill LAJ. Krebs Cycle reimagined: The emerging roles of succinate and itaconate as signal transducers. Cell (2018) 174(4):780–4. doi: 10.1016/j.cell.2018.07.030 30096309

[18] Mills EL Ryan DG Prag HA Dikovskaya D Menon D Zaslona Z. Itaconate is an anti-inflammatory metabolite that activates Nrf2 *via* alkylation of KEAP1. Nature (2018) 556(7699):113–7. doi: 10.1038/nature25986 PMC6047741 29590092

[19] Swain A Bambouskova M Kim H Andhey PS Duncan D Auclair K. Comparative evaluation of itaconate and its derivatives reveals divergent inflammasome and type I interferon regulation in macrophages. Nat Metab (2020) 2(7):594–602. doi: 10.1038/s42255-020-0210-0 32694786 PMC7378276

[20] Hooftman A Angiari S Hester S Corcoran SE Runtsch MC Ling C. The immunomodulatory metabolite itaconate modifies NLRP3 and inhibits inflammasome activation. Cell Metab (2020) 32(3):468–78.e7. doi: 10.1016/j.cmet.2020.07.016 32791101 PMC7422798

[21] Ren J Yu L Lin J Ma L Gao DS Sun N. Dimethyl itaconate inhibits neuroinflammation to alleviate chronic pain in mice. Neurochem Int (2022) 154:105296. doi: 10.1016/j.neuint.2022.105296 35121012

[22] Plunkett JA Yu CG Easton JM Bethea JR Yezierski RP. Effects of interleukin-10 (IL-10) on pain behavior and gene expression following excitotoxic spinal cord injury in the rat. Exp Neurol (2001) 168(1):144–54. doi: 10.1006/exnr.2000.7604 11170729

[23] Kriek N Groeneweg JG Stronks DL de Ridder D Huygen FJ. Preferred frequencies and waveforms for spinal cord stimulation in patients with complex regional pain syndrome: A multicentre, double-blind, randomized and placebo-controlled crossover trial. Eur J Pain (2017) 21(3):507–19. doi: 10.1002/ejp.944 27714945

[24] Ge J Yan Q Wang Y Cheng X Song D Wu C. IL-10 delays the degeneration of intervertebral discs by suppressing the p38 MAPK signaling pathway. Free Radic Biol Med (2020) 147:262–70. doi: 10.1016/j.freeradbiomed.2019.12.040 31883468

[25] Rempenault C Mielle J Schreiber K Corbeau P Macia L Combe B. #CXCR5/CXCL13 pathway, a key driver for migration of regulatory B10 cells, is defective in patients with rheumatoid arthritis. Rheumatol (Oxford) (2021) 61(5):2185–96. doi: 10.1093/rheumatology/keab639 34382069

[26] Shao Q Li Y Wang Q Zhao J. IL-10 and IL-1beta mediate neuropathic-pain like behavior in the ventrolateral orbital cortex. Neurochem Res (2015) 40(4):733–9. doi: 10.1007/s11064-015-1521-5 25617163

[27] Fonseca MM Davoli-Ferreira M Santa-Cecilia F Guimaraes RM Oliveira FFB Kusuda R. IL-27 counteracts neuropathic pain development through induction of IL-10. Front Immunol (2019) 10:3059. doi: 10.3389/fimmu.2019.03059 32047492 PMC6997342

[28] Iwasa T Afroz S Inoue M Arakaki R Oshima M Raju R. IL-10 and CXCL2 in trigeminal ganglia in neuropathic pain. Neurosci Lett (2019) 703:132–8. doi: 10.1016/j.neulet.2019.03.031 30904573

[29] De Souza DP Achuthan A Lee MK Binger KJ Lee MC Davidson S. Autocrine IFN-I inhibits isocitrate dehydrogenase in the TCA cycle of LPS-stimulated macrophages. J Clin Invest (2019) 129(10):4239–44. doi: 10.1172/JCI127597 PMC6763227 31483287

[30] Hu T Sun Q Gou Y Zhang Y Ding Y Ma Y. Salidroside alleviates chronic constriction injury-induced neuropathic pain and inhibits of TXNIP/NLRP3 pathway. Neurochem Res (2022) 47(2):493–502. doi: 10.1007/s11064-021-03459-y 34626306

[31] O'Neill LAJ Artyomov MN. Itaconate: the poster child of metabolic reprogramming in macrophage function. Nat Rev Immunol (2019) 19(5):273–81. doi: 10.1038/s41577-019-0128-5 30705422

[32] Marquez S Fernandez JJ Mancebo C Herrero-Sanchez C Alonso S Sandoval TA. Tricarboxylic acid cycle activity and remodeling of glycerophosphocholine lipids support cytokine induction in response to fungal patterns. Cell Rep (2019) 27(2):525–36.e4. doi: 10.1016/j.celrep.2019.03.033 30970255

[33] Liao ST Han C Xu DQ Fu XW Wang JS Kong LY. 4-octyl itaconate inhibits aerobic glycolysis by targeting GAPDH to exert anti-inflammatory effects. Nat Commun (2019) 10(1):5091. doi: 10.1038/s41467-019-13078-5 31704924 PMC6841710

[34] Sun X Zhang B Pan X Huang H Xie Z Ma Y. Octyl itaconate inhibits osteoclastogenesis by suppressing Hrd1 and activating Nrf2 signaling. FASEB J (2019) 33(11):12929–40. doi: 10.1096/fj.201900887RR PMC6902740 31490085

[35] Tian F Wang Z He J Zhang Z Tan N. 4-octyl itaconate protects against renal fibrosis *via* inhibiting TGF-beta/Smad pathway, autophagy and reducing generation of reactive oxygen species. Eur J Pharmacol (2020) 873:172989. doi: 10.1016/j.ejphar.2020.172989 32032597

[36] Li Q Lan X Han X Durham F Wan J Weiland A. Microglia-derived interleukin-10 accelerates post-intracerebral hemorrhage hematoma clearance by regulating CD36. Brain Behav Immun (2021) 94:437–57. doi: 10.1016/j.bbi.2021.02.001 PMC8058329 33588074

[37] Kakizaki M Watanabe R. IL-10 expression in pyramidal neurons after neuropathogenic coronaviral infection. Neuropathology (2017) 37(5):398–406. doi: 10.1111/neup.12386 28493345 PMC7167951

[38] Lobo-Silva D Carriche GM Castro AG Roque S Saraiva M. Balancing the immune response in the brain: IL-10 and its regulation. J Neuroinflamm (2016) 13(1):297. doi: 10.1186/s12974-016-0763-8 PMC5121946 27881137

[39] Dominguez-Andres J Novakovic B Li Y Scicluna BP Gresnigt MS Arts RJW. The itaconate pathway is a central regulatory node linking innate immune tolerance and trained immunity. Cell Metab (2019) 29(1):211–20.e5. doi: 10.1016/j.cmet.2018.09.003 30293776

[40] Yi Z Deng M Scott MJ Fu G Loughran PA Lei Z. Immune-responsive gene 1/Itaconate activates nuclear factor erythroid 2-related factor 2 in hepatocytes to protect against liver ischemia-reperfusion injury. Hepatology (2020) 72(4):1394–411. doi: 10.1002/hep.31147 PMC7702080 31997373

[41] Liang Y Chen Y Li L Zhang S Xiao J Wei D. Krebs Cycle rewired: Driver of atherosclerosis progression? Curr Med Chem (2021) 29(13):2322–33. doi: 10.2174/0929867328666210806105246 34365937

[42] Cordes T Lucas A Divakaruni AS Murphy AN Cabrales P Metallo CM. Itaconate modulates tricarboxylic acid and redox metabolism to mitigate reperfusion injury. Mol Metab (2020) 32:122–35. doi: 10.1016/j.molmet.2019.11.019 PMC6961711 32029222

[43] Sendra M Saco A Rey-Campos M Novoa B Figueras A. Immune-responsive gene 1 (IRG1) and dimethyl itaconate are involved in the mussel immune response. Fish Shellfish Immunol (2020) 106:645–55. doi: 10.1016/j.fsi.2020.07.034 32798695

[44] Marrocco A Frawley K Pearce LL Peterson J O'Brien JP Mullett SJ. Metabolic adaptation of macrophages as mechanism of defense against crystalline silica. J Immunol (2021) 207(6):1627–40. doi: 10.4049/jimmunol.2000628 PMC8428747 34433619

[45] Ryan DG O'Neill LAJ. Krebs Cycle reborn in macrophage immunometabolism. Annu Rev Immunol (2020) 38:289–313. doi: 10.1146/annurev-immunol-081619-104850 31986069

[46] Belosludtsev KN Belosludtseva NV Kosareva EA Talanov EY Gudkov SV Dubinin MV. Itaconic acid impairs the mitochondrial function by the inhibition of complexes II and IV and induction of the permeability transition pore opening in rat liver mitochondria. Biochimie (2020) 176:150–7. doi: 10.1016/j.biochi.2020.07.011 32721502

[47] Apryani E Ali U Wang ZY Wu HY Mao XF Ahmad KA. The spinal microglial IL-10/beta-endorphin pathway accounts for cinobufagin-induced mechanical antiallodynia in bone cancer pain following activation of alpha7-nicotinic acetylcholine receptors. J Neuroinflamm (2020) 17(1):75. doi: 10.1186/s12974-019-1616-z PMC7049212 32113469

[48] Wu HY Mao XF Tang XQ Ali U Apryani E Liu H. Spinal interleukin-10 produces antinociception in neuropathy through microglial beta-endorphin expression, separated from antineuroinflammation. Brain Behav Immun (2018) 73:504–19. doi: 10.1016/j.bbi.2018.06.015 29928964

[49] Peace CG O'Neill LA. The role of itaconate in host defense and inflammation. J Clin Invest (2022) 132(2):e148548. doi: 10.1172/JCI148548 35040439 PMC8759771

[50] Daniels BP Kofman SB Smith JR Norris GT Snyder AG Kolb JP. The nucleotide sensor ZBP1 and kinase RIPK3 induce the enzyme IRG1 to promote an antiviral metabolic state in neurons. Immunity (2019) 50(1):64–76.e4. doi: 10.1016/j.immuni.2018.11.017 30635240 PMC6342485

[51] Wang L He C. Nrf2-mediated anti-inflammatory polarization of macrophages as therapeutic targets for osteoarthritis. Front Immunol (2022) 13:967193. doi: 10.3389/fimmu.2022.967193 36032081 PMC9411667

[52] Zhang FH Sun YH Fan KL Dong XB Han N Zhao H. Protective effects of heme oxygenase-1 against severe acute pancreatitis *via* inhibition of tumor necrosis factor-alpha and augmentation of interleukin-10. BMC Gastroenterol (2017) 17(1):100. doi: 10.1186/s12876-017-0651-4 28836936 PMC5571505

[53] Speth C Joebstl B Barcova M Dierich MP. HIV-1 envelope protein gp41 modulates expression of interleukin-10 and chemokine receptors on monocytes, astrocytes and neurones. AIDS (2000) 14(6):629–36. doi: 10.1097/00002030-200004140-00001 10807185

[54] Yang J Jiang Z Fitzgerald DC Ma C Yu S Li H. Adult neural stem cells expressing IL-10 confer potent immunomodulation and remyelination in experimental autoimmune encephalitis. J Clin Invest (2009) 119(12):3678–91. doi: 10.1172/JCI37914 PMC2786785 19884657

